# Microfluidic Mechanoporation: Current Progress and Applications in Stem Cells

**DOI:** 10.3390/bios14050256

**Published:** 2024-05-17

**Authors:** Rubing Wang, Ziqi Wang, Lingling Tong, Ruoming Wang, Shuo Yao, Di Chen, Huan Hu

**Affiliations:** 1 Zhejiang University-University of Illinois Urbana-Champaign Institute (ZJU-UIUC Institute), International Campus, Haining 314400, China; rubing.21@intl.zju.edu.cn; 2 Center for Regeneration and Cell Therapy of Zhejiang University-University of Edinburgh Institute (ZJU-UoE Institute), Zhejiang University School of Medicine, Zhejiang University, Hangzhou 310003, China; ziqiw.20@intl.zju.edu.cn (Z.W.); lingling.21@intl.zju.edu.cn (L.T.); 3 Zhejiang University-University of Edinburgh Institute (ZJU-UoE Institute), International Campus, Zhejiang University, Haining 314400, China; ruoming.21@intl.zju.edu.cn (R.W.); shuo.22@intl.zju.edu.cn (S.Y.); 4 Center for Reproductive Medicine, The Second Affiliated Hospital, School of Medicine, Zhejiang University, Hangzhou 310003, China; 5 Dr. Li Dak Sum & Yip Yio Chin Center for Stem Cell and Regenerative Medicine, Zhejiang University, Hangzhou 310003, China; 6 National Key Laboratory of Biobased Transportation Fuel Technology, Haining 314400, China

**Keywords:** microfluidic, intracellular delivery, mechanoporation, stem cells

## Abstract

Intracellular delivery, the process of transporting substances into cells, is crucial for various applications, such as drug delivery, gene therapy, cell imaging, and regenerative medicine. Among the different approaches of intracellular delivery, mechanoporation stands out by utilizing mechanical forces to create temporary pores on cell membranes, enabling the entry of substances into cells. This method is promising due to its minimal contamination and is especially vital for stem cells intended for clinical therapy. In this review, we explore various mechanoporation technologies, including microinjection, micro–nano needle arrays, cell squeezing through physical confinement, and cell squeezing using hydrodynamic forces. Additionally, we highlight recent research efforts utilizing mechanoporation for stem cell studies. Furthermore, we discuss the integration of mechanoporation techniques into microfluidic platforms for high-throughput intracellular delivery with enhanced transfection efficiency. This advancement holds potential in addressing the challenge of low transfection efficiency, benefiting both basic research and clinical applications of stem cells. Ultimately, the combination of microfluidics and mechanoporation presents new opportunities for creating comprehensive systems for stem cell processing.

## 1. Introduction

Intracellular delivery is an important technique in molecular and cell biology research, which introduces biomaterials into cells for investigating the regulation of gene expression, functions of genes of interest, protein–protein interactions, the sub-cellular localization of proteins, and for genome editing and gene therapy [1]. In recent decades, many effective methods have been established to achieve intracellular transport with higher efficiency.

Cell transfections with DNA can be divided into transient transfections and stable transfections according to the expression duration of the exogenous biomaterials in cells. In transient transfections, exogenous DNA does not integrate into the host chromosome and only lasts for several days because of the dilution upon cell divisions [2]. On the contrary, the stable transfection of exogenous DNA could be integrated into host genomes and then express target genes or proteins constitutively in the cells depending on the regulatory sequences applied to drive the expression [3]. Currently, traditional transection methods can be divided into three categories [4]: (1) biological transfection methods mediated by viral vectors, including lentivirus, adenovirus, and adeno-associated viruses [5,6,7,8,9]; (2) chemical transfection methods using different transfection media, such as calcium phosphate [10], liposome [11], and cationic polymers [12,13]; and (3) mechanical methods to achieve transfections through the disturbance or destruction of cell membranes, such as electroporation [14], microinjection [15,16], the gene gun method [17], and acoustic hole effect-mediated transfections [18,19].

The transfection of biomaterials into cells has greatly improved our understanding of gene function and regulation. However, the above methods have their own shortcomings that limit the applications of transfections into different types of cells, especially for stem cells. For a viral vector-mediated transfection, although it exhibits high transfection efficiency with the continuous and stable expression of exogenous genes [20], there are safety concerns, because the insertion site of the viral vectors into the host genome is uncertain. This uncertain gene integration may cause the activation of proto-oncogenes, the inactivation of oncogenes, RNA splicing, and gene fusion, thus posing a risk of carcinogenesis [21,22,23,24]. For chemical transfection methods, although the transfection efficiency has been improved after liposome modification [25], it is still expensive, and the transfection efficiency is still low for stem cells. Mechanical transfection methods have been successfully applied to different cell types with high efficiency, including stem cells [15,18,26,27]. However, they require specific equipment and complex operation processes, which significantly increase cell mortality [16,18,26,28]. Therefore, we expect an intracellular transfection method that is suitable for many cell types, with high transfection efficiency, cell biosafety, economy, ease of operation, and so on. Emerging microfluidic technology [29,30] is promising due to its low solvent consumption, low counter dose, small cell-like volume, and a relatively high transfection efficiency and cell survival rate. It can be applied to a wide range of applications. In addition, the microfluidic environment is close to the diameter of cells, which is conducive to single-cell research and even in situ visual observations and real-time monitoring [31,32,33,34,35,36].

Although there exist several excellent reviews on mechanoporation [37,38,39], there is no review particularly targeting stem cells. We particularly selected mechanoporation approaches that are integrated with microfluidic chips for intracellular delivery to stem cells with high throughput and a low dead rate. In this review, we introduce different methods based on microfluidic transfections, including microinjection [31,32,40,41,42,43], micro/nanoneedle arrays [44,45,46,47,48], cell squeezing based on mechanical confinement [33,34,49], and cell squeezing based on hydrodynamic manipulation [35,36]. Furthermore, we briefly introduce the current progress for applying microfluidic methods in stem cell research, highlighting the advantages and limitations.

## 2. Microfluidic-Based Mechanoporation

**Figure 1:**
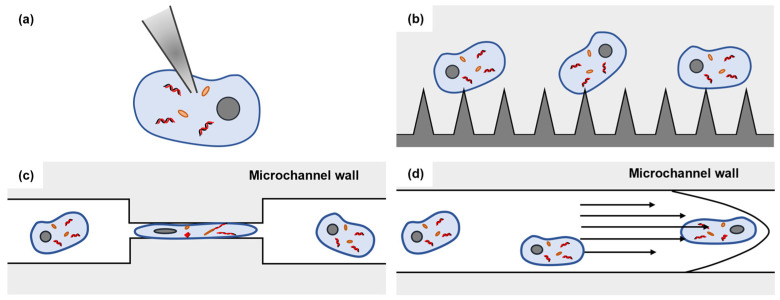
Microfluidic-based mechanoporation methods. (**a**) Microinjection; (**b**) micro/nanoneedle arrays; (**c**) cell squeezing based on mechanical confinement; (**d**) cell squeezing based on hydrodynamic manipulation.

As a critical step in microfluidic cell transfections, membrane disruption-based intracellular delivery methods drew a lot of attention from researchers [50,51] and can be classified into electroporation [52,53,54,55,56,57], optoporation [58,59,60,61,62], magnetoporation [63,64,65], acoustoporation [66,67,68,69], and mechanoporation [34,48,70,71,72,73] based on pore creation mechanisms. While each technique above possesses its own set of advantages, it is important to note that all except for mechanoporation rely on an external energy field to disrupt the cell membrane. However, this dependence on external energy fields can potentially impact the biological function and viability of the cells being manipulated [74,75,76]. Therefore, this review focuses on the mechanoporation techniques that are independent of an external energy field. We will discuss four different mechanoporation techniques that employ only mechanical structures without causing severe damage to cell membranes. Based on the different microfluidic device structures, microfluidic-based mechanoporation methods can be classified as microinjection (Figure 1a), micro/nanoneedle arrays (Figure 1b), cell squeezing based on mechanical confinement (Figure 1c), and cell squeezing based on hydrodynamic manipulation (Figure 1d). All these methods exhibit advantages, such as high transfection efficiency, high throughput, ease of handling, and high cell viability [34,48,70,71,72,73].

### 2.1. Microinjection

Microinjection, ever since its inception in the previous century [77], has remained a commonly utilized method for single-cell transfections due to both its straightforward concept and ease of manipulation. By inserting a glass micropipette into specific positions of individual cells [78], almost any cargo can be successfully delivered into the cells via microinjection. This versatile technique finds applications in various areas, including in vitro fertilization and nuclear transfer for cloning [79]. As one of the traditional microinjection schemes, the AFM (atomic force microscopy)-based microinjection is adopted for the precise intracellular delivery to single cells by functionalizing antibodies to the AFM probe [38,80] or through the hollow AFM cantilever [81]. Benefiting from a size of 200–300 nm and a high aspect ratio structure, the AFM tip can penetrate the cell and adhere to the substrate with proper force and cause little or no damage to the cell membrane [39]. However, one major limitation of this approach is its low throughput and limited suitability for suspended types of cells, since it depends on the surface adhesion property of cells. The introduction of microfluidic techniques provides a platform to better manipulate all types of cells for microinjections, improving the intracellular delivery throughput and enabling suspension cell transfections [31,32,40,41,42,43].

**Figure 2:**
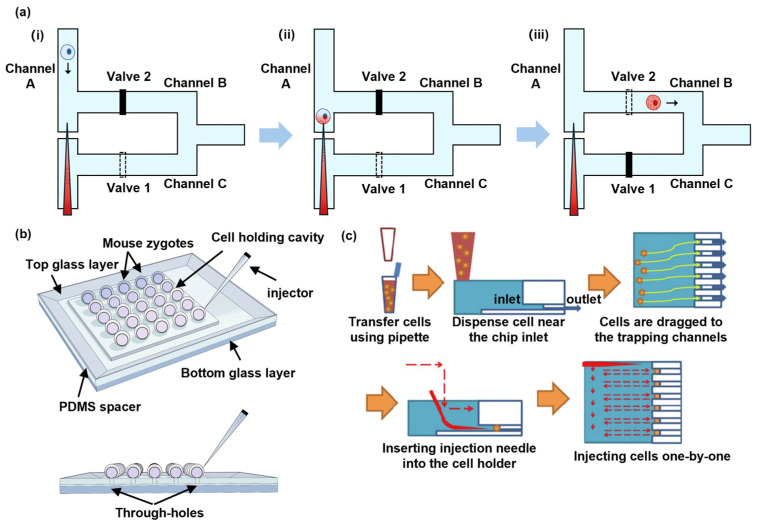
Microinjection. (**a**) A schematic illustration of the microfluidic-based single-cell microinjection system. (**i**) Cells are driven from Channel A to Channel B by fluid stream. (**ii**) Cells are transfected by immobilized microneedle. (**iii**) Cells are driven from Channel B to Channel C by fluid stream. Reprinted and modified from Ref. [31]. (**b**) The vacuum-based cell-holding device for single-cell immobilization. Reprinted and modified from Ref. [40]. (**c**) A workflow illustration of the automated quantitative microinjection platform. Reprinted and modified with permission from Ref. [32].

By integrating microinjection and microfluidic techniques, Adamo and Jensen proposed a microneedle-immobilized microfluidic microinjection device [31]. As shown in Figure 2(ai), in this device, single cells were driven by fluid streams from channel A to channel B and transfected by immobilized microneedles while valve 1 was opened and valve 2 was closed. After cell transfection, the cells were driven by fluid streams from channel B to channel C by closing valve 1 and opening valve 2. The experimental results showcase an approximate throughput of 1 cell in 5 min, conducted with HeLa cells [31]. To enhance the throughput of the microinjection system, Liu and Sun presented a vacuum-based cell-holding device for single-cell immobilization and applied this device to a mouse zygote microinjection [40]. In this study, mouse zygotes were immobilized into arrays of 5 × 5 through-holes (Figure 2b), making cell capture and immobilization easier and allowing for the transfection of a total amount of 200 s at a speed of 9 cells/min, substantially improving the throughput of traditional microinjection methods. The experimental results demonstrate the progression of zygotes into the blastocyst stage after microinjection, providing evidence for the claim that the microneedle-immobilized microfluidic microinjection device would not affect embryo survival and development [40].

To improve injection automation for effective transfections [32,41,42,43], an automated quantitative microinjection platform was developed by Chow et al., showcasing the ability to deliver precise quantities of materials into cells [32]. By immobilizing cells in a microfluidic chip and injecting a certain amount of substances through an injection pressure- and time-controlled micropipette to cells one by one (Figure 2c), this microinjection platform achieved a precise single-cell microinjection. This microinjection platform, which was applied to human foreskin fibroblast cells, achieved about 80% transfection efficiency and 82.1% cell viability. However, this microinjection still suffers from low throughput, limiting its application to larger amounts of cells.

### 2.2. Micro/Nanoneedle Arrays

Compared with microinjections with a single pipette, the integration of micro/nanoneedles into microfluidic-based devices could achieve high-throughput and efficient single-cell transfections. Microfabrication techniques can fabricate different micro/nanoneedle array structures in a straightforward and convenient fashion [44,45].

**Figure 3:**
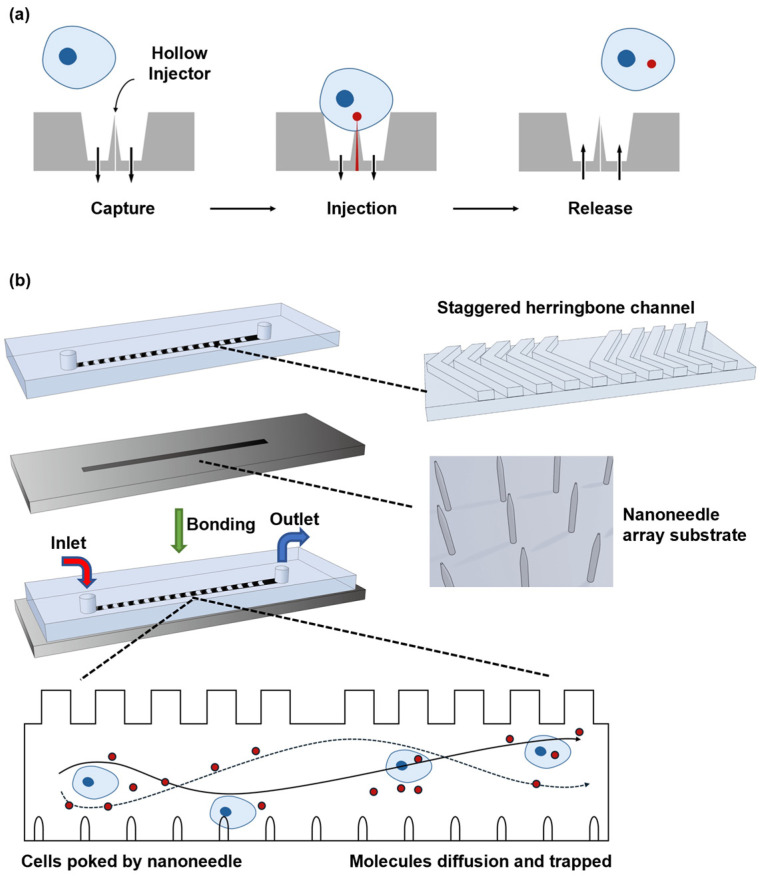
Micro/nanoneedle arrays. (**a**) A schematic of the microneedle arrays for the single-cell capture site. The arrows denote the flow direction and magnitude. Reprinted and modified from Ref. [46]. (**b**) A schematic illustration of the PDMS-based nanoneedle arrays. Reprinted and modified from Ref. [48].

Zhang et al. proposed a microfluidic microneedle device with massively parallel microinjector arrays, enabling a superhigh throughput microinjection [46]. As shown in Figure 3a, this device operates by attracting the cell onto the hollow penetrator during aspiration-based captures. Subsequently, exogenous cargos are injected into the cell through the resulting membrane pore before the cells are released by a positive aspiration flow. Each microinjector in the microneedle array incorporates a hollow penetrator with a sub-micron tip with a base of approximately 1–2 μm in diameter. In this device, the negative and positive aspiration flows ensure the minimal force required for cell capture and penetration, since they allow for the minimal stress of the sub-micro tip to penetrate the cell membrane. Moreover, the massively parallel microinjector array, which refers to 100 × 100 capture sites, realizes an ultrahigh throughput microinjection. Further experimental results exhibit a transfection efficiency of approximately 93% at a flow rate of 40 μL/min using an immortalized human T lymphocyte cell line-applied propidium iodide dye [47]. In the case of delivering a green fluorescent protein plasmid, efficiency rates of 82% in the primary human T cells were achieved, with over 87% cell viability. Overall, this microfluidic microneedle device demonstrates high efficiency and throughput capabilities for microinjections, showcasing its potential in various cellular transfection applications [47].

Furthermore, Huang et al. devised a microfluidic nanoneedle device including a silicon nanoneedle array along with the staggered herringbone channel design [48]. In this design, as depicted in Figure 3b, a PDMS structure featured a channel on its top surface, which was composed of periodically staggered herringbone grooves. By incorporating two asymmetrically shifted groups of staggered herringbone grooves, this configuration facilitated the chaotic mixing of the substances introduced through the inlet port. Unlike microinjections which directly deliver exogenous cargos into the cells, exogenous cargos are diffused into the cell after the cells collide with the nanoneedle array and then form pores on the cell membrane. The experimental results, achieved with human embryonic kidney cells, demonstrate transfection efficiency of over 20% and cell viability exceeding 95% while transfected with GFP-expressing plasmids [48].

### 2.3. Cell Squeezing Based on Mechanical Confinement

In response to the drawbacks of microinjections and micro/nanoneedle arrays potentially causing irreversible damage to cell membranes, researchers developed mechanical confinement-based cell-squeezing strategies. In these methods, the cell membrane undergoes rapid mechanical deformation when passing through a microfluidic constriction smaller than its size, leading to the formation of transient holes. These holes are recoverable, meaning that the damage caused to the cell membrane is almost negligible.

**Figure 4:**
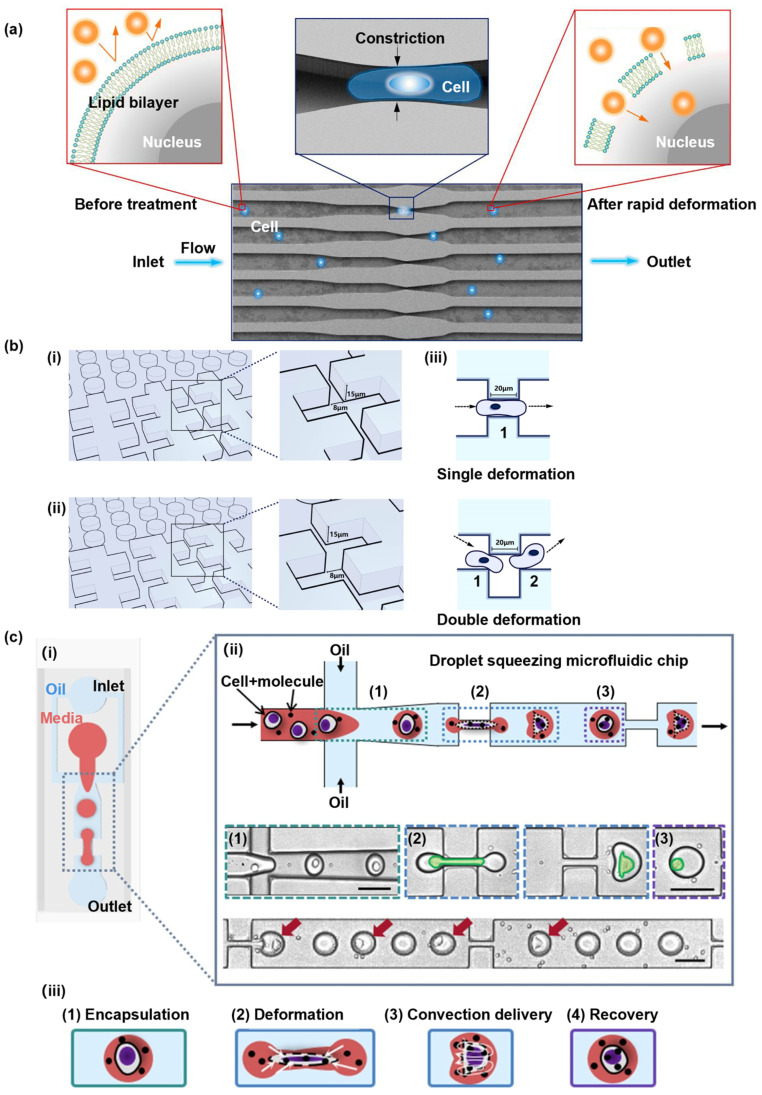
Cell squeezing based on mechanical confinement. (**a**) An illustration of the delivery hypothesis, whereby the rapid deformation of a cell, as it passes through a microfluidic constriction, generates transient membrane holes. Reprinted and modified with permission from Ref. [33]. (**b**) Two designs of a microfluidic cell deformation device. (**i**,**ii**) Schemata of the two designs. (**iii**) An illustration of the cell-squeezing process in two different cell deformation devices. Reprinted and modified from Ref. [34]. (**c**) The droplet squeezing platform design. (**i**) A schematic of a droplet squeezing microfluidic device. (**ii**) An illustration of the working flow of the platform and high-speed microscope images that show the three stages of the cell in the platform; (1) encapsulation, (2) deformation, and (3) restoration. (**iii**) A schematic diagram illustrating the delivery mechanism of droplet squeezing, owing to a convection-based cargo transport. Reprinted and modified with permission from Ref. [49].

Sharei et al. [33] demonstrated cell squeezing based on the mechanical confinement method for cell delivery, in which multiple cells undergo mechanical squeezing simultaneously when passing through parallel micro-constriction channels. Figure 4a clearly demonstrates that when cells were subjected to a constriction channel narrower than their size, a temporary disruption of the cell membrane was observed. Transient pores were generated, which promoted intracellular delivery based on the diffusion of biomaterials into the cell. This method achieves an average throughput rate of 20,000 cells per second, which is significantly higher than that of the microfluidic device that employs the aforementioned microinjections and micro/nanoneedle arrays, exhibiting about 75% delivery efficiency and a maximum of 95% cell viability while transferring blue-labeled 3 kDa dextran molecules into HeLa cells [33]. By introducing key transcription factors (Oct4, Sox2, c-Myc, and Klf-4) required for stem cell pluripotency into human fibroblast cells [82], Sharei et al. implemented cell reprogramming. The identification of transformed colonies expressing embryonic stem cell markers reveals the morphological transformation in human fibroblast cells and the effect on gene expression, providing more possibilities for cell therapy and regenerative medicine.

To further enhance cell delivery efficiency, Modaresi et al. introduced a microfluidic platform to perform double cell deformation [34]. Figure 4b illustrates two microfluidic device designs, one allowing for single deformation (Figure 4(bi)) and the other allowing for double deformation (Figure 4(bii)). In the case of the first design, cells were subjected to continuous paralleled constrictions, which were 20 μm in length and 8 μm in width, permitting single deformation. Conversely, the second device translated one side of the narrow channel in the first design to create staggered squeeze constrictions with an 8 μm gap, enabling double transformation. The experiments showed that the double-deformation approach resulted in the higher delivery efficiency of biomaterials into cells compared to the single deformation method while applying human adipose-derived stem cells that were transfected with Dex-FITC. This device, which allows for cell double deformation, is superior for delivering small-sized exogenous materials, achieving an 85% delivery efficiency and improving cell viability to 95%, while maintaining a higher throughput. Furthermore, it did not induce the cell apoptosis associated with the single-deformation method.

Joo et al. proposed a microfluidic device for droplet mechanoporation, where cells encapsulated with biomolecules in one droplet are transported through multiple constrictions to prevent cell damage and increase cell viability [49]. This device, as illustrated in Figure 4(ci), comprises two parts: the droplet generator and the cell-squeezing sections. By injecting oil through separated inlet channels and utilizing a droplet generation technique [83], cell-biomolecule-encapsulated droplets are formed, leading to an increasing localized concentration of biomolecules that enhances cell delivery efficiency. As cells traverse through the constrictions within droplets, they experience a synergistic effect of convection and diffusion-mediated transport. This dynamic combination enables the efficient delivery of biomolecules through the cell membrane (Figure 4(ciii)). This method maximizes transfection efficiency, with a remarkable 98% achieved in a high throughput of 1 million cells per minute, and provides a minimum cell survival rate of 80%. Moreover, since each droplet carries the required cargo and most of the microchannel is occupied by carrier oil, significantly less cargo is utilized, minimizing the risk of clogging issues.

### 2.4. Cell Squeezing Based on Hydrodynamic Manipulation

As explained in Section 2.3, although cell-squeezing-type microfluidic devices based on channel confinement can achieve high-throughput cell deformation, they often suffer from cell membrane damage caused by the narrow channels as well as device failure due to clogging. To overcome these challenges, researchers explored hydrodynamic forces to control single cells stretching or squeezing in a microchannel. During this process, transient pores are generated on the cell membrane, facilitating the delivery of exogenous material through a blend of fluid convection and diffusion. The risk of microchannel clogging and cell lysis is significantly reduced, since the cells are not squeezed using constriction channels. Hydrodynamic techniques for creating transient nanopores offer several advantages, such as a simple design, inexpensive equipment requirements, and the capability to achieve the high-throughput intracellular delivery of diverse biomaterials into a broad spectrum of cells.

**Figure 5:**
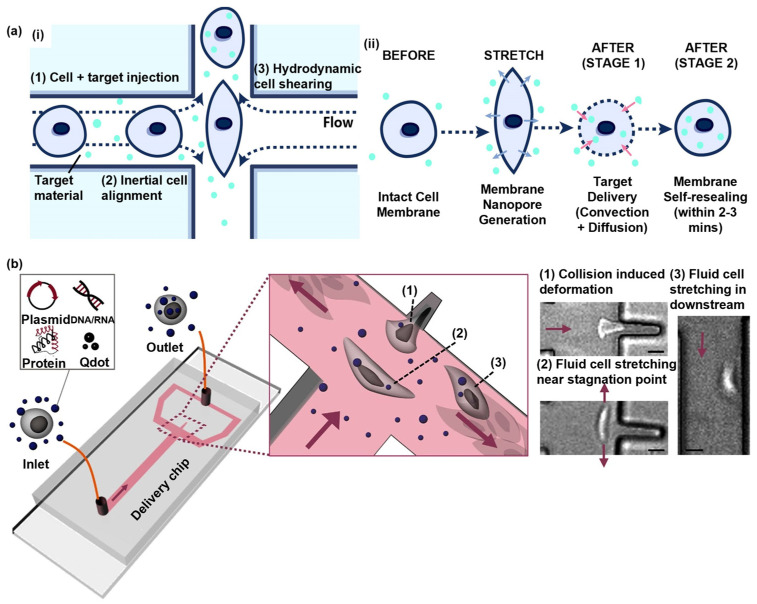
Cell squeezing based on hydrodynamic manipulation. (**a**) Hydroporator. (**i**) A schematic illustration of the design and operating principles of the vector-free intracellular delivery system. (**ii**) An illustration of the delivery mechanism. Reprinted and modified from Ref. [35]. (**b**) A schematic of the fluidic cell-stretching platform. High-speed microscope images showing the three stages of cell deformation (1)~(3). All arrows indicate the main flow direction (scale bars: 15 μm). Reprinted and modified with permission from [36]. Copyright (2020), American Chemical Society.

Kizer et al. reported a hydrodynamic manipulation-based cell-stretching approach [35] that effectively eliminated the possibility of the device clogging observed in earlier designs (Figure 5(ai)). In this proposed system, transient pores were formed on the cell membrane through the rapid hydrodynamic shearing of the cells, and the stagnation point, at which the transient fluid velocity is zero, is generated by two fluids with the same velocity but in opposite directions. As the cells approached the cross-section, they experienced hydrodynamic stretching and reached the maximum degree of deformation at the stagnation point, leading to the generation of transient membrane nanopores (Figure 5(aii)). Due to the rapid exchange of cytosol and external fluids across the cell membrane, this method facilitated convection-based intracellular delivery during the cell-stretching process (Figure 5(aii)), showing that transfection efficiency increased as the flow rates (i.e., Reynolds number) increased, while the cell viability decreased as the Reynolds number increased. Therefore, a suitable Reynolds number is the key to balancing transfection efficiency and cell viability. As a result, the experimental results demonstrate the successful delivery of DNA into various cell types, such as K-562, MDA-MB-231, HeLa cells, and so on, with a transfection efficiency of over 90%, an approximately 80% cell viability, and a remarkable throughput of over 1,600,000 cells per minute when the Reynolds number equals to 189. This approach showcased its effectiveness in achieving efficient delivery while maintaining cell viability by carefully controlling the Reynolds number to optimize the performance.

To simplify operations and improve the efficiency of material transportation, J. Hur et al. introduced a hydrodynamic manipulation-based cell-stretching intracellular delivery platform [36]. The device contained a T-shaped microchannel equipped with a small cavity, which provided the intrinsic inertial flow to deform the cell passing by. In the T-shaped microchannel, cells are exposed to elongational flows, enabling their lateral migration toward the center of the channel through intrinsic inertial flow. This mechanism allows for the uniform stretching of cells. As illustrated in Figure 5b, each cell hit into the cavity due to the force of elongational flows, leading to a collision-induced deformation. Subsequently, the cells were released from the cavity and reached maximum deformation at the stagnation point and then underwent slight cell stretching downstream while moving to the outlet. This study applied the cell delivery mechanism, which involves a mixture of a convection and diffusion-based solution exchange across the cell membrane during the cell-stretching and -recovering processes. J. Hur et al. achieved a knockdown of the ITGA1 gene by delivering siRNA into Hela cells using this cell-stretching device. Cells subjected to this microfluidic cell-stretching device exhibited a near-complete suppression of the ITGA1 gene expression, with a knockdown efficiency of 97% [36], indicating the tremendous potential of this technique in genetic editing. Overall, this intracellular delivery platform offers several advantages, including a high delivery efficiency of up to 98%, a high throughput of up to 1 million cells per minute, simplicity in operation, low material costs, and the ability to deliver various cell types and biomaterials.

### 2.5. Summary

In this section, four different microfluidic mechanoporation methods were discussed, and each of them has its advantages and disadvantages (Table 1). Due to the accurate cargo delivery by inserting the micropipette into cells, microinjections ensure uniform transfections and achieve high transfection efficiency. Nevertheless, this method has limitations, such as low throughput rates and a high cost, which arises from the need for specialized expertise and expensive devices. Compared with microinjections, micro/nanoneedle arrays offer advantages, such as higher throughput and ease of use, as they allow for the simultaneous perforation of multiple cells. However, the manufacturing process of micro/nanoneedle arrays is usually complex and costly. Also, the effectiveness of this approach is dependent on the cell type utilized, as optimal results can typically be achieved with adherent cells. As for cell squeezing based on mechanical confinement, this has the advantage of high throughput and the ability to achieve intracellular delivery for a wide range of cells on the one hand and has the disadvantages of device clogging and non-uniform transfections. Instead of transforming cells by narrow channels in the microfluidic device, cell squeezing based on hydrodynamic manipulation avoids the issue of device clogging and maintains high throughput at the same time. Meanwhile, it also has the drawback of non-uniform transfections.

## 3. Application of Mechanoporation in Stem Cells

Stem cells are critical for the homeostasis of tissues and organs. Upon each cell division, the daughter cells can maintain as stem cells (self-renewal) or initiate a differentiation program for functional cells to replace the old, dead, or damaged cells. Understanding how the self-renewal and differentiation of stem cells is balanced is critical for clinical applications. Exogeneous gene expression and genome editing are both crucial for not only understanding how stem cells are regulated but also for the application of gene-edited cells for clinical purposes, highlighting the importance of delivering biomaterials into stem cells.

They possess self-renewal and differentiation capabilities, thus holding broad prospects for basic research and clinical applications. Human embryonic stem cells (hESCs) were first isolated in 1998, and since then, several adult stem cells, induced pluripotent stem cells (iPSCs), have been isolated as important models for basic research [84,85,86]. Due to the unique self-renewal and differentiation potential of stem cells, stem cell therapy has the potential to treat diseases, such as heart disease and type I diabetes [87,88].

Intracellular transfection technology is a crucial step for applications of stem cells, since it can introduce exogenous genes or small molecule drugs into stem cells, thereby changing the transcriptome state and physiological functions of stem cells for different purposes. For example, transfecting CRISPR-related components into cells can be used for the gene editing of stem cells [89],, and transfecting small molecules into stem cells can be used to mark them, which can be applied to stem cell therapy [90,91].

### 3.1. Comparison of Different Transfection Methods in Stem Cells

**Figure 6:**
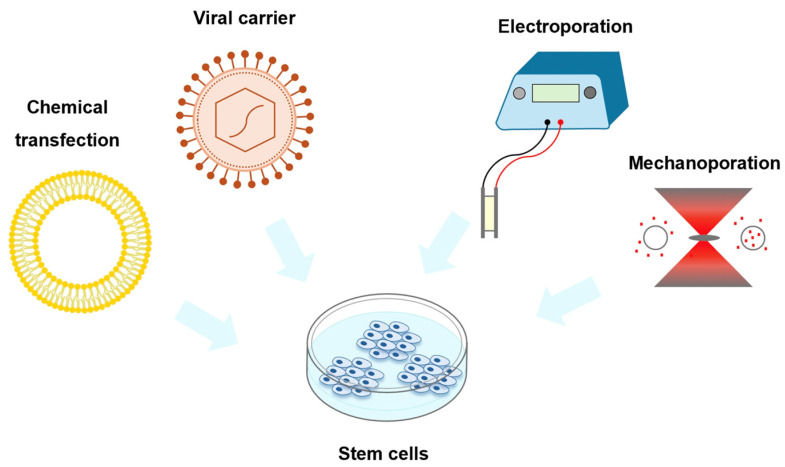
Schematic diagram of different stem cell-transfection methods.

In order to conduct stem cell research and application, many technical problems must be solved, one of which is how to efficiently deliver external genes or drugs into stem cells through intracellular transfection. To improve delivery efficiency, scientists have established different means to optimize the delivery scheme (Figure 6). Common stem cell-transfection techniques include chemical transfections, electroporation, and viral vector transfections [92,93,94,95]. Although these methods can successfully introduce exogenous genes or small molecules into stem cells, there are many limitations (Table 2).

Chemical transfections suffer from the cytotoxicity of the transfection material and the low transformation efficiency in primary cells and stem cells [96]. Electroporation applies an electric field across the cells to perforate the cell membrane, achieving higher transfection efficiency, although specialized equipment and manual handling are required. In addition, electrical cell perforation causes a high cell death rate and low stability [56,97,98]. Viral vector-based transfections can achieve high-efficiency transfections, but there are biological safety issues, and the random insertion of viral vectors into the genome may lead to a disruption of local genes, resulting in unexpected risks, such as cell death [99]. It is essential to apply the transfection method with high transfection efficiency, a high survival rate, easy operation, low cost, and the large-scale operation of cells for different types of stem cells, enabling the application of stem cells and stem cell-derived functional cells for regenerative medicine.

As mentioned above, mechanoporation is a new type of transfection method, which promotes cell deformation through mechanical force, resulting in increased membrane permeability. This strategy improves the incorporation of therapeutic substances, such as DNA, RNA, and drugs, into the cells. Mechanoporation increases the transfection efficiency and improves the survival rate of cells, thereby facilitating the research of stem cells [36,90,91,100]. Therefore, mechanoporation may represent the best strategy for various fields of stem cell research.

### 3.2. Application of Mechanoporation in Stem Cells

Transfection efficiency and cell viability are critical for applying stem cells for clinical applications. Due to the characteristics of less cell damage, a high cell survival rate, and high transfection efficiency, mechanoporation has shown great prospects in the clinical treatment of regenerative medicine.

**Figure 7:**
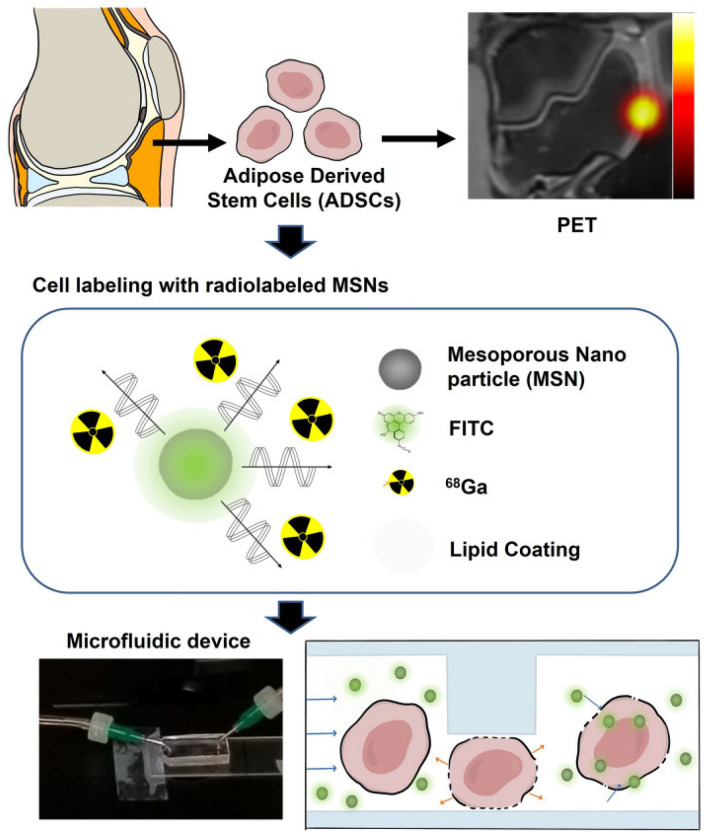
Experimental scheme of mechanoporation, which enables the rapid and efficient radiolabeling of adipose tissue-derived stem cells (ADSCs) for PET imaging. Reprinted with permission from Ref. [90].

Adipose tissue-derived stem cells (ADSCs) are one of the well-studied stem cells for clinical applications. As shown in Figure 7, Jung et al. employed mechanically perforated ADSCs for rapid labeling for PET/MRI imaging [90]. The patient’s own stem cells can be used to repair or regenerate damaged joint tissue. In this context, these transplanted cells need to be labeled with in vivo molecular imaging tools to distinguish them from the host cells. In the follow-up treatment, it is necessary to monitor and observe the implantation, survival, migration, and differentiation activities to achieve the purpose of predicting the therapeutic effects. Jung et al. applied mechanical compression to transport iron oxide nanoparticles and 18F-FDG into ADSCs for subsequent identification by PET/MRI. The labeling process can be completed in a very short time during the operation, and the labeling efficiency is similar to that achieved by passive incubation for 30 min. The detection of labeled cells found that, compared with unlabeled cells, the survival rate of labeled cells reached 94%, and there was no increase in long-term toxicity, and even DNA damage was lower than that of passive incubation methods.

On the other hand, stem cells cultured in vitro are an important research system for applications in regenerative medicine. Intracellular transfection technology can be used to introduce specific regulatory proteins or modified enzymes to explore the regulatory mechanisms governing the self-renewal and differentiation of stem cells. Intracellular transfection combined with gene editing technology has also been widely used in stem cell research. Therefore, the difficult-to-transfect feature has become a major challenge in stem cell research.

Chung et al. used fluidic cell mechanoporation to successfully transfect primary human stem cells with plasmid DNA. Cell viability, after mechanoporation, was significantly higher than electroporation [36]. Garcia et al. demonstrated a novel microfluidic device for the successful transfection of mRNA into human primary T cells, natural killer (NK) cells, and CD34+ hematopoietic stem and progenitor cells (HSPCs) via volume exchange for convective transfections (VECTs) [100]. In addition to the role of intracellular transport, studies have found that mechanical stretching can promote the reprogramming efficiency of fibroblasts to functional cells, even skipping the process of reprogramming into stem cells. When suspension cells pass through a narrow microfluidic channel, the nucleus undergoes a rapid extrusion. This reversible nuclear deformation process can significantly reduce the methylation level of histone H3K9 and DNA, thereby improving chromatin accessibility. Finally, it promotes the reprogramming efficiency of fibroblasts to neurons [101].

**Figure 8:**
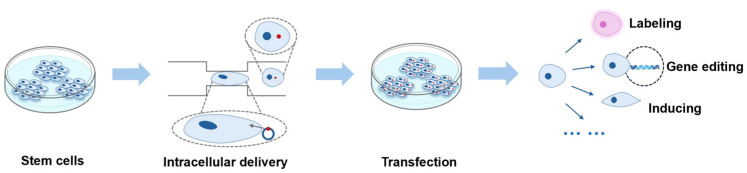
Transfection of the stem cell and its main application.

Through these stem cell studies, scientists can better understand the properties and functions of stem cells and apply them in various fields of regenerative medicine and disease treatment. Mechanoporation technology is an important tool for stem cell research, which helps scientists better utilize the potential of stem cells to achieve more effective therapeutic effects (Figure 8). Although this technology may still have some limitations and challenges at present, we believe that with continuous development and updating, mechanical perforation technology will exert more potential and make greater contributions in applying stem cells in regenerative medicine.

## 4. Outlook and Conclusions

Microfluidic mechanoporation, as an emerging intracellular delivery approach, offers huge potential in stem cell applications due to the following advantages.

First, mechanoporation offers the precious feature of no contamination or cytotoxicity, which is critical for stem cell study or therapy. For stem cell therapy, there must be product control, meaning the gene-edited stem cells should be exactly edited as we expect. But other intracellular delivery methods can introduce potential complications, such viral vectors, chemicals, and electrical stimulations, which may bring gene mutation or contamination. Mechanoporation only uses mechanical forces to disrupt cell membranes introducing no chemicals or viruses. In addition, a microfluidic chip can achieve the whole function of stem cell therapy by integrating sample processing, such as filtering and purification, gene editing using mechanoporation, quality checks, etc., in one personalized chip, holding promising applicational potential. Even single-cell manipulation, gene editing, and quality checks can all be realized in one microfluidic chip.

Second, microfluidic mechanoporation has great versatility, simplicity, and low fabrication costs. It allows for different types of cargos, such as nucleic acids, small molecules, and proteins to be delivered to various types of cells. It does not require any external energy sources, such as an electrical field or an acoustic field, and is straightforward to use. The fabrication of a microfluidic mechanoporation device is quite mature using conventional soft lithography or standard silicon microfabrication.

Third, microfluidic mechanoporation can offer high delivery efficiency and high cell viability, which is critical for gene editing and stem cell therapy. Low transfection efficiency in stem cells is one of the main bottlenecks for both basic research and clinic applications. Human pluripotent stem cells, including embryonic stem cells and induced pluripotent stem cells, possess the capacity to differentiate into different kinds of functional cells for cell therapy. However, the transfection efficiency is extremely low compared with cancerous cell lines. Furthermore, the transfection of large-size plasmid DNA or proteins is also inefficient. Cell state, extracellular components, and other cell membrane characteristics may “protect” stem cells from taking exogenous materials, making the traditional methods inefficient. Mechanoporation may substantially increase the transfection efficiency, because it stretches cells by mechanical forces. High transfection efficiency and the potential for delivering large cargoes will trigger both the mechanistic research and translational applications of stem cells.

Microfluidic mechanoporation technology still has a few challenges to overcome, including a lack of precise pore size control, a relatively low transfection efficiency for some cell types, and potential cell damages, which could be solved with a further understanding of the mechanoporation mechanisms and optimized device and operation parameters.

With a fast-growing interest in gene-editing-relevant applications, we can foresee more research development and commercialization in microfluidic mechanoporation technology toward more ideal technology that features high throughput, low costs, high cell viability, excellent reliability, and a more straightforward utilization.

## Figures and Tables

**Table 1:** Comparison of four microfluidic mechanoporation methods.

Microfluidic Mechanoporation Method	Advantages	Disadvantages	Throughput (Cells/min)	Cell Viability
Microinjection	Uniform transfection	High cost	<100	82.5%
	High transfection efficiency	Low throughput rates		
Micro/nanoneedle arrays	Higher throughput than microinjection	Complex and costly manufacturing	>10,000	95%
	Ease of use	Dependent on the cell type		
Cell squeezing based on mechanical confinement	High throughput	Device clogging	>1,000,000	95%
	Suitable for a wide range of cells	Non-uniform transfections		
Cell squeezing based on hydrodynamic manipulation	High throughput	Non-uniform transfections	>1,600,000	80%
	No device clogging			

**Table 2:** Comparison of different stem cell-transfection methods.

Transfection Method	Advantages	Disadvantages
Chemical Transfection	Simple operation	Low transfection efficiency
		Toxicity of the biomaterial
Viral Carrier	High transfection efficiency	Low biosecurity
		Instability due to random insertion
Electroporation	High transfection efficiency	Low stability
	High mortality rate	High cost due to difficult operation
Mechanoporation	High transfection efficiency	
	Capable of mass operation	
	High cell survival rate	
	High stability	
